# Development of a Simple *In Vitro* Assay to Assess Digestion of the Extracellular Matrix of the Human Pancreas by Collagenase Enzyme Blends

**DOI:** 10.1177/0963689718779778

**Published:** 2018-06-28

**Authors:** Rebecca M. Spiers, Sarah E. Cross, Helen L. Brown, Paul A. Bateman, Rebecca H. Vaughan, Stephen J. Hughes, Paul R. V. Johnson

**Affiliations:** 1 Islet Transplant Research Group, Nuffield Department of Surgical Sciences, University of Oxford, Level 6, John Radcliffe Hospital, Oxford, UK; 2 Oxford Center for Diabetes, Endocrinology and Metabolism (OCDEM), University of Oxford, Churchill Hospital, Oxford, UK

**Keywords:** collagenase, extracellular matrix, islet, islet isolation

## Abstract

Despite huge advances in the field of islet transplantation over the last two decades, current islet isolation methods remain suboptimal, with transplantable yields obtained in less than half of all pancreases processed worldwide. Successful islet isolation is dependent on the ability of collagenase-based enzyme blends to digest extracellular matrix components at the islet–exocrine interface. The limited availability of donor pancreases hinders the use of full-scale islet isolations to characterize pancreas digestion by different enzyme components or blends, or allow the influence of inter-pancreatic variability between donors to be explored. We have developed a method that allows multiple enzyme components to be tested on any one pancreas. Biopsies of 0.5 cm^3^ were taken from seven standard (age ≥45) and eight young (age ≤35) pancreases. Serial cryosections were treated with Serva collagenase, neutral protease (NP), or the two enzymes together at clinically relevant concentrations. Following digestion, insulin and either collagen IV or laminin-α5 were detected by immunofluorescent labeling. Protein loss at the islet–exocrine interface was semi-quantified morphometrically, with reference to a control section. Differential digestion of the two proteins based on the enzyme components used was seen, with protein digestion significantly influenced by donor age. Treatment with collagenase and NP alone was significantly more effective at digesting collagen IV in the standard donor group, as was the NP mediated digestion of laminin-α5. Collagenase alone was not capable of significantly digesting laminin-α5 in either donor group. Combining the two enzymes ameliorated the age-related differences in the digestion of both proteins. No significant differences in protein loss were detected by the method when analyzed by two independent operators, demonstrating the reproducibility of the assay. The development of this simple yet reproducible assay has implications for both enzyme batch testing and identifying inter-donor digestion variability, while utilizing small amounts of both enzyme and human tissue.

## Introduction

Pancreatic islet transplantation is an effective treatment for reversing life-threatening hypoglycemia unawareness in patients with type 1 diabetes^1,2^. However, despite the improvement in clinical outcomes, human islet isolation remains a variable process, with transplantable yields obtained in fewer than 50% of pancreases processed worldwide^1,3,4^.

The success of islet isolation is largely dependent on the ability of commercially available collagenase-based enzyme blends to digest extracellular matrix (ECM) components, specifically at the islet–exocrine interface, to liberate free and intact islets^5^. One reason for the unpredictability of the pancreas digestion phase of the human islet isolation procedure is the variability of commercially available collagenases^6^. Islet isolation success is also influenced by donor factors, such as age^7–9^. In addition, our knowledge of the structure of the islet–exocrine interface and the nature of the substrate that we are trying to digest at this interface remains poor. As successful human islet isolation depends on effective separation of islets from their surrounding exocrine tissue, more detailed knowledge of the composition of the peri-islet pancreatic matrix on which collagenase is acting is vital^10,11^. The islet–exocrine interface is composed of an interstitial matrix and a specialized basement membrane (BM), which in addition to its structural role is thought to influence β-cell insulin secretion and survival^12–14^. BMs generally consist of a collagen IV network, which forms a scaffold into which the other BM components, including members of the laminin family, integrate in a highly organized, supramolecular architecture^15^. The human islet BM exists as a unique duplex structure, with each layer differing in composition. A vascular BM associated with the islet capillary is present in addition to an endocrine BM integral to the islet itself^16^. Laminin-α5 is found in both layers and can thereby be used as a marker for the integrity of the duplex BM^16^. A greater understanding of the protein composition of the matrix at the islet–exocrine interface and the susceptibility of each component to digestion will enable the development of novel, tailored enzyme blends, which will help to optimize islet isolation outcomes from the full range of donor pancreases.

Due to the limited availability of donor pancreases, it is difficult to use the full-scale islet isolation procedure to conduct detailed experiments characterizing pancreas digestion by different enzyme components or different enzyme blends, and to determine how digestion is influenced by inter-pancreatic variability between donors. Instead, what is required is a simple method that enables multiple enzyme components and combinations of components to be tested on any one pancreatic sample. We describe the development of such a research method.

## Materials and Methods

### Sampling Technique

With appropriate consent and ethical approval, human pancreases from standard (≥45 years old; *n* = 7) and young (≤35 years old; *n* = 8) multi-organ donors were procured for islet isolation (Table 1). Tissue samples of 0.5 cm^3^ were taken from the head of the pancreas and snap-frozen in liquid nitrogen. Frozen tissue sections (10 µm thickness) were prepared using a Cryostat (Leica, Milton Keynes, UK) and collected onto Superfrost Plus glass slides (Thermo-Fisher Scientific, Loughborough, UK), before storage at –25°C until use.

**Table 1.** Donor and pancreas characteristics.

	Young donors ≤ 35 years (n=8)	Standard donors ≥ 45 years (n=7)	*P* value
Age, years (mean ± SEM)	30 ± 1	54±2	<0.001
Age, years (median (range))	31 (25–35)	53 (46–60)	<0.001
Gender (M: F)	6:2	5:2	0.876
BMI (mean ± SEM)	26 ± 1	27 ± 1	0.29
CIT, h (mean ± SEM)	5.6 ± 0.5	7.0 ± 1.4	0.089
Donor type (DBD: DCD)	5:3	7:0	0.07

BMI, body mass index; CIT, cold ischemia time; DBD, donation after brain death; DCD, donation after cardiac death; SEM, standard errors of the mean

### Enzymatic Treatment and Immunolabeling of Tissue Sections

Pancreas cryosections were thawed in HBSS (Lonza, Basel, Switzerland) before undergoing incubation for 5 min at 37°C with HBSS (control), collagenase NB1 alone, neutral protease NB alone (both Serva, Heidelberg, Germany), or a combination of the two enzymes at clinically relevant concentrations (collagenase 7.2 PZU/ml, neutral protease 0.14 DMCU/ml). Following treatment, enzymatic activity was quenched by placing slides in cold running water. Sections were fixed with 2.5% paraformaldehyde (Sigma, Poole, UK) and then blocked with 10% normal goat serum or 5% normal swine serum (Vector Laboratories, Peterborough, UK), for laminin-α5 and collagen IV staining, respectively, for 30 min at room temperature (r.t). Sections then underwent double immunofluorescent labeling. Incubation with primary antibodies – polyclonal guinea pig anti-insulin (1:100; Dako, Ely, UK), and either monoclonal mouse anti-laminin-α5 (1:100; Abcam, Cambridge, UK) or polyclonal goat anti-collagen IV (1:100; BioRad, Oxford, UK) – was for 1.5 h at r.t. Sections were then incubated with secondary antibodies for 30 min at r.t. Texas Red conjugated goat anti-guinea pig (1:100; Vector Laboratories) and AMCA conjugated horse anti-mouse (1:100; Vector Laboratories) were used for laminin-α5 detection, or AlexaFluor 594 conjugated donkey anti-guinea pig (1:500; Jackson Immunoresearch, PA, USA) and AlexaFluor 405 conjugated donkey anti-goat (1:100; Abcam) were used for collagen IV detection. Slides were mounted with ProLong Diamond Antifade mounting media without DAPI (Thermo-Fisher Scientific) and cured overnight at 4°C. For sections not undergoing enzyme treatment, slides were treated as above but without the initial digestion step – that is, sections were thawed, fixed, and blocked, then incubated with primary antibodies as above for collagen IV and laminin- α5, or with polyclonal goat anti-collagen VI (1:50; BioRad) and monoclonal mouse anti-perlecan (1:100; Santa Cruz Biotechnology, Dallas, TX, USA). Secondary antibodies were as above, or: AlexaFluor 405 conjugated donkey anti-goat (1:100; Abcam) for collagen VI detection; and fluorescein conjugated horse anti-mouse (1:100; Vector Laboratories) for perlecan detection.

Non-specific binding of the secondary antibodies was tested by omitting primary antibodies. Negative control sections were incubated with normal host IgG (Santa Cruz Biotechnology) in place of the primary antibodies at appropriate concentrations to test for non-specific binding of primary antibodies.

### Quantitative Analysis of Digestion

Islets were identified by immunofluorescent labeling of insulin. A minimum of ten islets were imaged per donor, per treatment, using an Axioskop 40 microscope and Axiovision software (Carl Zeiss, Jena, Germany) in 8-bit format. The corresponding field of protein staining (laminin-α5 or collagen IV) was also imaged in the same format. For all images captured, the image settings, including the exposure time, remained constant with respect to the control. The images were imported into Image J, where the immunolabeled protein that encapsulated the islet and penetrated into the intra-islet region was semi-quantified morphometrically using a purpose-built semi-automated macro. Briefly, the outline of the islet was selected by drawing around the insulin-positive region. The images were converted to 32-bit before the area of the islet was automatically recorded. This region of interest was then superimposed onto a 32-bit version of the corresponding protein image, and the area and intensity of protein staining within this islet region was measured. A threshold value was set to a standard 25, which removed any positive regions that were likely to have occurred due to background staining. The threshold area of the protein staining was then normalized against the whole islet area to take into account islet size, and then multiplied by the mean signal threshold intensity. The data generated from the treatment conditions were normalized to the control, to give a percentage loss from the control.

### Assay Reproducibility

To demonstrate the reproducibility of the technique, complete image sets from five randomly selected donors for each protein were independently analyzed by an additional researcher, on a different computer system, using the semi-automated macro described above.

### Statistical Analysis

Digestion data were analyzed using mixed linear modeling to determine both enzyme component and donor age effects on protein digestion. Data are expressed as mean ± standard error of the mean (SEM). Wilcox paired sign rank test was used to determine whether there was a significant difference in the results obtained from the two independent researchers. All statistical analyses were performed in R (R Core Team, Vienna, Austria). For all analyses performed, *P* < 0.05 was considered significant.

## Results

Studies were performed on tissue biopsies obtained from both standard (≥45 years) and younger (≤35 years) donor pancreases accepted for clinical islet isolation (Table 1). The mean donor age was 30 ± 1 years and 54 ± 2 years, in the young and standard groups respectively. Body mass index (BMI), cold ischemia time (CIT), and gender ratio were statistically matched between the two groups. Retrieval of the organs either occurred after donor cardiac death (donation after cardiac death; DCD) or after donor brain death (donation after brain death; DBD). Although three pancreatic biopsies in the younger group were obtained from DCD donors, compared with no donors of this type in the standard donor group, this did not reach statistical significance (*P* = 0.07).

### Quantitative Analysis of Digestion

**Figure 1.**
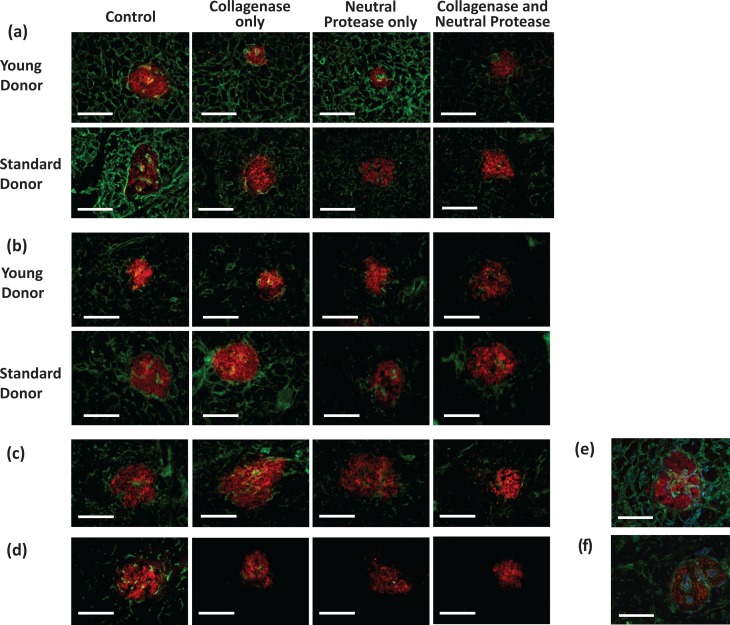
Immunofluorescence labeling of ECM protein digestion at the islet–exocrine interface of young and standard donors. Representative images of pancreas sections following treatment with HBSS (control), collagenase, neutral protease, or a combination of both enzymes for 5 min. Digestion of (a) collagen IV and (b) laminin-α5 (both green) is seen around and within the islet (red, insulin labeling of β-cells). Loss of signal for both proteins was significantly greater in the standard donor group than the young group when treated with neutral protease. There was no significant loss of laminin-α5 when treated with collagenase alone in either group, though this treatment was effective at digesting collagen IV in both donor groups. The loss of collagen IV by collagenase was more effective in standard donors. The combination of enzymes ameliorated the significant age-related digestion differences in both proteins. Representative images to show the effect of each enzyme component alone and in combination on digestion of (c) collagen VI (green) and (d) perlecan (green) both around and within the islet (red, insulin labeling of β-cells). Representative images showing the localization of each ECM protein around and within the islet. Triple immunofluorescent staining for: (e) collagen IV (green), laminin-α5 (blue), and insulin (red); (f) collagen VI (green), perlecan (blue), and insulin (red). Scale bars = 100 μm.

The developed slide-based digestion assay was able to successfully demonstrate differential digestion of peri-islet ECM proteins collagen IV (Figure 1(a,e)), laminin-α5 (Figure 1(b,e)), collagen VI (Figure 1(c,f)), and perlecan (Figure 1(d,f)) when utilizing individual components of clinical islet isolation enzyme blends. The assay was also capable of determining enzyme component-based donor age-related differences in the digestion of these proteins (Figure 1(a,b)). Loss of matrix proteins following time-controlled digestion was detected by the reduction in signal intensity post-immunofluorescent staining for the protein of interest, following normalization to a control slide.

**Figure 2.**
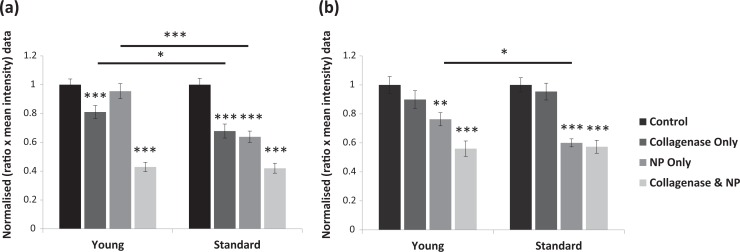
Quantitative analysis of collagen IV and laminin-α5 digestion. The area and intensity of immunofluorescent staining for (a) collagen IV and (b) laminin-α5 was normalized to the control, post-digestion with specific enzyme components, and the efficiency of digestion was compared between two donor age groups. Independent use of neutral protease led to significantly more loss of both collagen IV and laminin-α5 in the standard donor group than in the younger group. There was no significant loss of laminin-α5 when treated with collagenase alone in either group. Collagenase significantly digested collagen IV in both donor groups, but was more effective on standard donors. The combination of enzymes ameliorated the significant age-related digestion differences in both proteins. Data represent the mean ± SEM from *n* = 6 each donor group (collagen IV) and *n* = 7 each donor group (laminin-α5). *P <0.05, **P<0.01, ***P<0.001 vs control, or as indicated by solid bars. NP, neutral protease.

When sections were treated for 5 min with collagenase alone there was a 32 ± 5% reduction in collagen IV in the standard donor group (Figure 2(a), *P* < 0.001), but only a 19 ± 5% loss of collagen IV in the young donor group (*P* < 0.001). Despite the significant collagenase-mediated loss of collagen IV in both groups, there was significantly more loss in the standard donor group (*P* < 0.05). Neutral protease (NP) was ineffective at digesting collagen IV in young donors (4 ± 5% loss compared with control; NS) whereas significant digestion occurred in the standard donor group (36 ± 4% loss compared with control; *P* < 0.001). NP was therefore significantly more effective at digesting collagen IV in the standard donor group (*P* < 0.001). Despite the enhanced resistance of collagen IV to digestion in the young donors when treated with collagenase or NP independently, combining the two enzymes ameliorated these significant differences and led to a 57 ± 4% and 58 ± 3% reduction in collagen IV in the young and standard donor groups respectively (both *P* < 0.001, compared with respective control).

When treated with collagenase alone, digestion of laminin-α5 was not significant in either donor group (loss of 10 ± 6% and 5 ± 6% for young and standard groups respectively, as compared with respective controls; both NS). However, laminin-α5 was significantly digested by NP in both donor groups (loss of 24 ± 5% and 40 ± 3% for young and standard groups respectively, as compared with respective controls; *P* < 0.01 and *P* < 0.001), though digestion was significantly greater in the standard donors (*P* = 0.05). The significant age-related differences in digestion were lost when collagenase and NP were combined. This combined treatment led to a loss of 44 ± 5% and 43 ± 4% in the amount of laminin-α5 for the young and standard groups, respectively (both *P* < 0.001, compared with respective control).

No non-specific binding of either primary or secondary antibodies was detected by the control staining.

### Assay Reproducibility

**Figure 3.**
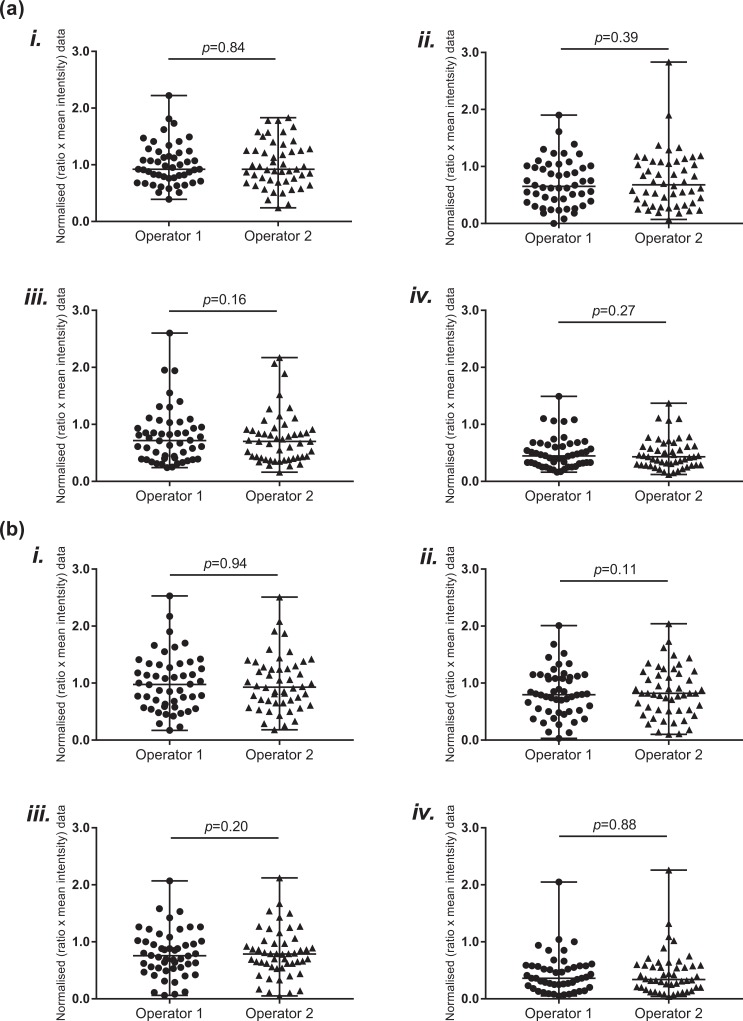
Assay reproducibility. To assess assay reproducibility, complete image sets for both (a) collagen IV and (b) laminin-α5, from five randomly selected donors, were independently analyzed by a second operator. Data represent the ratio of positive protein labeling to that of the insulin stained region. This ratio was then multiplied by the mean intensity of the labeling. All treatment data were normalized to the respective control. There was no significant variation in the measurements obtained from control (i), collagenase only (ii), neutral protease (NP) only (iii), or collagenase and NP (iv). Graphs display all data points, as measured by both operators (*n* = 5 donors for each protein), along with the median and range.

To assess the reproducibility of the assay, complete image sets obtained from five donors for each protein were analyzed by an independent operator, as per the method described. There was no significant variation in the data obtained from the image analysis between the two operators for either collagen IV (Figure 3(a)) or laminin-α5 (Figure 3(b)).

## Discussion

A simple, slide-based digestion assay was developed in order to characterize digestion of the pancreatic matrix by collagenase and NP enzymes used in clinical islet isolation. We were able to use this assay to investigate the influence of donor age and the role of specific components of enzyme blends in the digestion of ECM proteins comprising the islet–exocrine interface.

As donor pancreases are presently the only source of islets for clinical transplantation, optimizing human islet isolation remains a research priority. In particular, understanding human pancreas digestion at the molecular level is paramount. In order to do this, it is important to characterize the pancreatic matrix in detail, and to develop targeted enzyme blends that optimally digest the profile of matrix proteins found in different donor types. Human pancreases are subject to a wide range of different donor variables, including BMI and past medical and social histories. In addition, variables during procurement and transport of the pancreas, for example donation type (DBD vs. DCD), quality of organ perfusion, and CIT, will influence the success of islet isolation^7,9^. Donor age is one of the most reported factors affecting isolation outcomes, and may influence pancreatic composition and structure, in turn impacting on the effectiveness of digestion by collagenase enzyme blends.

This novel technique, first proposed by our group in 2007^17^, allows characterization of the effect of collagenase and supplementary protease enzymes on digestion of specific substrates. It enables testing of multiple enzyme batches and components on any one human pancreas, in addition to the analysis of collagenase components individually and in combination, using pancreases from the full range of donors accepted for islet transplantation. The reproducibility of the technique was demonstrated, as analysis of digestion can be compared using multiple slides from the same donor pancreas sample, and between different researchers using the same slides.

We have shown that there is variability in the digestion efficiencies of collagenase and NP on individual components of the islet–exocrine interface, which is further influenced by the age of the pancreatic donor. The normalization of data to a control tissue section, which was treated with HBSS alone, eliminates any potential interference from endogenous proteolytic enzymes and ensures that any digestion seen is as a result of the exogenously added enzymes only, as all data are normalized to the control.

We chose to study the digestion of two human islet BM proteins – collagen IV and laminin-α5. Collagen IV is the predominant structural component of the islet BM, forming a scaffold into which the other BM components, including laminins and heparan sulfate proteoglycans (HSPGs), integrate to form an organized supramolecular architecture^15^. The human islet BM contains a variety of laminin isoforms^16^. Laminins are known to be involved in numerous cell survival and function signaling pathways^14,15,18,19^. The laminin-α5 isoform is present in both layers of the duplex BM^16^ and so is an ideal marker to study BM integrity, along with collagen IV due to its role as the major structural BM component. By using the assay we have demonstrated that digestion of peri-islet laminin-α5 is dependent on NP, and is significantly more effective in digesting this protein in donors ≥45 years of age, when compared with a young donor group of ≤35 years of age. The digestion of collagen IV was also significantly influenced by the age of the donor. Although NP was ineffective at digesting collagen IV in younger donors, significant digestion occurred in the standard donor group. Collagenase digestion of collagen IV was also significantly greater in the standard donor group. The ability of the technique to detect such important differences substantiates its usefulness and practicality.

There have been previous attempts to develop simple assays for batch-testing of enzymes by incubating small samples of pancreatic tissue with different enzymes at 37°C, followed by sampling of digested and undigested tissue and isolation of small numbers of islets^20^. An alternative approach involves division of the pancreas into sections, each of which is infused via the pancreatic duct with a different enzyme, followed by a small-scale islet isolation procedure using each section. The main limitations to these methods are the requirement for greater volumes of enzyme and the necessity for larger pieces of pancreatic tissue, in addition to the additional time and labor required. There is also a limit to the number of enzymes and combinations that can be tested on any one pancreatic sample, as it is not possible to obtain large biopsies from pancreases received for clinical isolation. In contrast, our slide-based assay allows high-throughput, and because it uses frozen sections of tissue there is no requirement for fresh samples. Small tissue samples (0.5 cm^3^) can be taken from pancreases received for clinical islet isolation without compromising efficient distension of the pancreas with enzyme during the islet isolation procedure, and these can be stored long term. This not only provides an important resource, enabling the testing of enzyme batches on the same pancreas, therefore eliminating errors caused by inter-pancreatic variability during testing, but also supports the ongoing investigation of pancreatic structure, as demonstrated by these data. The identification of donor-related differences in the digestion of ECM proteins will aid in the development of novel, tailored enzyme blends specific to different donor types. This will be greatly beneficial in increasing islet yields from younger human donors, as effective isolation of islets from such donors presents an extreme challenge when utilizing current isolation blends^9,21^.

However, it is important to note that the slide-based assay does not replicate delivery of digestion enzymes into the pancreas by ductal infusion. Indeed, it is well known that efficient delivery of collagenase enzyme blends to the islet–exocrine interface using retrograde intraductal administration is crucial for optimal large-scale pancreas digestion and release of well-cleaved but intact islets^22^. The use of cryosections of pancreatic tissue in this assay may lead to increased exposure of the ECM surrounding the islet to the enzyme solution, which may not occur in the actual human islet isolation procedure. However, the simplicity and speed of our simple method allows for the rapid, simultaneous evaluation of enzyme batches using samples from a wide range of donor types, which would not be possible to do using standard islet isolation protocols. This method distinguishes between optimal, partial, and no digestion and can be used as an important evaluation tool for assessing multiple enzymes and enzyme combinations on any one pancreas. Based on the results from these “screening” tests, enzyme blends can then be selectively up-scaled to be tested within the full islet isolation procedure.

## Conclusions

This novel, slide-based digestion assay allows for the assessment of peri- and intra-islet ECM digestion by specific components of commercially available tissue digestion enzymes, as well as determining the influence of donor factors on protein digestibility. As the assay utilizes small amounts of both enzyme and human tissue, it is an ideal technique for batch-testing of enzymes, or for investigating variations in the digestion of the pancreatic ECM between donor types, such as the young and standard donor, which will facilitate the development of tailored enzyme blends for islet isolation.

## References

[1] Johnson PR Jones KE Pancreatic islet transplantation. Semin Pediatr Surg. 2012;21(3):272–280. 22800980 10.1053/j.sempedsurg.2012.05.012

[2] Leitao CB Tharavanij T Cure P Pileggi A Baidal DA Ricordi C Alejandro R Restoration of hypoglycemia awareness after islet transplantation. Diabetes Care. 2008;31(11):2113–2115. 18697903 10.2337/dc08-0741 PMC2571057

[3] Goto M Eich TM Felldin M Foss A Kallen R Salmela K Tibell A Tufveson G Fujimori K Engkvist M Korsgren O Refinement of the automated method for human islet isolation and presentation of a closed system for in vitro islet culture. Transplantation. 2004;78(9):1367–1375. 15548977 10.1097/01.tp.0000140882.53773.dc

[4] O’Gorman D Kin T Murdoch T Richer B McGhee-Wilson D Ryan E Shapiro AM Lakey JR The standardization of pancreatic donors for islet isolation. Transplant Proc. 2005;37(2):1309–1310. 15848705 10.1016/j.transproceed.2004.12.087

[5] Kin T Johnson PR Shapiro AM Lakey JR Factors influencing the collagenase digestion phase of human islet isolation. Transplantation. 2007;83(1):7–12. 17220782 10.1097/01.tp.0000243169.09644.e6

[6] Barnett MJ Zhai X LeGatt DF Cheng SB Shapiro AM Lakey JR Quantitative assessment of collagenase blends for human islet isolation. Transplantation. 2005;80(6):723–728. 16210957 10.1097/01.tp.0000174133.96802.de

[7] Lakey JR Warnock GL Rajotte RV Suarez-Alamazor ME Ao Z Shapiro AM Kneteman NM Variables in organ donors that affect the recovery of human islets of Langerhans. Transplantation. 1996;61(7):1047–1053. 8623183 10.1097/00007890-199604150-00010

[8] Nano R Clissi B Melzi R Calori G Maffi P Antonioli B Marzorati S Aldrighetti L Freschi M Grochowiecki T Socci C Secchi A Di Carlo V Bonifacio E Bertuzzi F Islet isolation for allotransplantation: Variables associated with successful islet yield and graft function. Diabetologia. 2005;48(5):906–912. 15830183 10.1007/s00125-005-1725-3

[9] Hanley SC Paraskevas S Rosenberg L Donor and isolation variables predicting human islet isolation success. Transplantation. 2008;85(7):950–955. 18408573 10.1097/TP.0b013e3181683df5

[10] Hughes SJ Clark A McShane P Contractor HH Gray DW Johnson PR Characterisation of collagen VI within the islet–exocrine interface of the human pancreas: Implications for clinical islet isolation? Transplantation. 2006;81(3):423–426. 16477230 10.1097/01.tp.0000197482.91227.df

[11] Van Deijnen JH Van Suylichem PT Wolters GH Van Schilfgaarde R Distribution of collagens type I, type III and type V in the pancreas of rat, dog, pig and man. Cell Tissue Res 1994;277(1):115–121. 8055531 10.1007/BF00303087

[12] Miao G Zhao Y Li Y Xu J Gong H Qi R Li J Wei J Basement membrane extract preserves islet viability and activity in vitro by up-regulating alpha3 integrin and its signal. Pancreas. 2013;42(6):971–976. 23587851 10.1097/MPA.0b013e318287cfe0

[13] Pinkse GG Bouwman WP Jiawan-Lalai R Terpstra OT Bruijn JA de Heer E Integrin signaling via RGD peptides and anti-beta1 antibodies confers resistance to apoptosis in islets of Langerhans. Diabetes. 2006;55(2):312–317. 16443762 10.2337/diabetes.55.02.06.db04-0195

[14] Wang RN Rosenberg L Maintenance of beta-cell function and survival following islet isolation requires re-establishment of the islet–matrix relationship. J Endocrinol. 1999;163(2):181–190. 10556766 10.1677/joe.0.1630181

[15] Yurchenco PD Basement membranes: cell scaffoldings and signaling platforms. Cold Spring Harb Perspect Biol. 2011;3(2):a004911. 10.1101/cshperspect.a004911 PMC3039528 21421915

[16] Virtanen I Banerjee M Palgi J Korsgren O Lukinius A Thornell LE Kikkawa Y Sekiguchi K Hukkanen M Konttinen YT Otonkoski T Blood vessels of human islets of Langerhans are surrounded by a double basement membrane. Diabetologia. 2008;51(7):1181–1191. 18438639 10.1007/s00125-008-0997-9

[17] Hughes SJ Gray DWR Clark A PRV J Development of an assay for the detailed study of collagenase-digestion within the islet–exocrine interface of the human pancreas. Xenotransplantation. 2007;14:485–485.

[18] Belkin AM Stepp MA Integrins as receptors for laminins. Microsc Res Tech. 2000;51(3):280–301. 11054877 10.1002/1097-0029(20001101)51:3<280::AID-JEMT7>3.0.CO;2-O

[19] Choong FJ Freeman C Parish CR Simeonovic CJ Islet heparan sulfate but not heparan sulfate proteoglycan core protein is lost during islet isolation and undergoes recovery post-islet transplantation. Am J Transplant. 2015;15(11):2851–2864. 26104150 10.1111/ajt.13366

[20] Johnson PR Van Suylichem PT Roberts DL Vos-Scheperkeuter GH White SA Van Schilfgaarde R London NJ A simple in vitro method for evaluating the efficacy of different batches of crude Clostridium histolyticum collagenase for islet isolation. Transplant Proc. 1995;27(6):3284–3285. 8539957

[21] Meier RP Sert I Morel P Muller YD Borot S Badet L Toso C Bosco D Berney T Islet of Langerhans isolation from pediatric and juvenile donor pancreases. Transpl Int. 2014;27(9):949–955. 24890668 10.1111/tri.12367

[22] Gray DW McShane P Grant A Morris PJ A method for isolation of islets of Langerhans from the human pancreas. Diabetes. 1984;33(11):1055–1061. 6437895 10.2337/diab.33.11.1055

